# Projecting malaria elimination in Thailand using Bayesian hierarchical spatiotemporal models

**DOI:** 10.1038/s41598-023-35007-9

**Published:** 2023-05-13

**Authors:** Chawarat Rotejanaprasert, Saranath Lawpoolsri, Patiwat Sa-angchai, Amnat Khamsiriwatchara, Chantana Padungtod, Rungrawee Tipmontree, Lynette Menezes, Jetsumon Sattabongkot, Liwang Cui, Jaranit Kaewkungwal

**Affiliations:** 1 Department of Tropical Hygiene, Faculty of Tropical Medicine, Mahidol University, Bangkok, Thailand; 2 Center of Excellence for Biomedical and Public Health Informatics (BIOPHICS), Faculty of Tropical Medicine, Mahidol University, Bangkok, Thailand; 3 Division of Vector Borne Diseases, Department of Disease Control, Ministry of Public Health, Nonthaburi, Thailand; 4 Division of Infectious Diseases and Internal Medicine, Department of Internal Medicine, University of South Florida, Tampa, USA; 5 Mahidol Vivax Research Unit, Faculty of Tropical Medicine, Mahidol University, Bangkok, Thailand

**Keywords:** Diseases, Health care

## Abstract

Thailand has set a goal of eliminating malaria by 2024 in its national strategic plan. In this study, we used the Thailand malaria surveillance database to develop hierarchical spatiotemporal models to analyze retrospective patterns and predict *Plasmodium falciparum* and *Plasmodium vivax* malaria incidences at the provincial level. We first describe the available data, explain the hierarchical spatiotemporal framework underlying the analysis, and then display the results of fitting various space–time formulations to the malaria data with the different model selection metrics. The Bayesian model selection process assessed the sensitivity of different specifications to obtain the optimal models. To assess whether malaria could be eliminated by 2024 per Thailand’s National Malaria Elimination Strategy, 2017–2026, we used the best-fitted model to project the estimated cases for 2022–2028. The study results based on the models revealed different predicted estimates between both species. The model for *P. falciparum* suggested that zero *P. falciparum* cases might be possible by 2024, in contrast to the model for *P. vivax*, wherein zero *P. vivax* cases might not be reached. Innovative approaches in the *P. vivax*-specific control and elimination plans must be implemented to reach zero *P. vivax* and consequently declare Thailand as a malaria-free country.

## Introduction

Thailand has successfully reduced indigenous malaria transmission, the cases contracted locally dropped from 150,000 cases in 2000 to 24,850 cases in 2015, with a morbidity rate of 0.38 per 1000 population^1^. Annual malaria-related mortality dropped significantly from 38,046 in 1949 to only 13 reported deaths in 2019 despite the resurgence of multidrug-resistant malaria over the decades^2^. Thailand’s last recorded 2836 indigenous cases in 2020 suggest that the designation of a malaria-free country by WHO is within reach^2^. In 2021, Thailand became one of 8 new countries eligible to eliminate malaria by 2025^2^, and the country’s national malaria control program has set a goal of eliminating malaria by 2024 in its national strategic plan.

A deeper understanding of the epidemiology of malaria in the last few foci in the country is essential for targeted monitoring and timely elimination. Prediction modeling using spatiotemporal statistics as part of an integrated malaria management system could help refine the analysis, allowing for micro-level precision and informing the development of effective elimination strategies^3^. As we noted in our previous work, developing these quantitative models is essential for monitoring and forecasting large-scale spatiotemporal processes of malaria^4^. When using a disease mapping framework, selecting appropriate linear predictors remains critical, especially when data involve spatial and temporal structures. Many methods (e.g., variable selection, model averaging, and transformation selection) have been proposed and utilized to achieve these goals (see examples in^5–7^). In certain conditions, the model selection procedure may be more relevant than simply using variable selection^5^.

In this study, we used the Thailand malaria surveillance database to develop hierarchical spatiotemporal models to analyze retrospective patterns and predict *Plasmodium falciparum* and *Plasmodium vivax* malaria incidences at the provincial level. We first describe the available data, explain the hierarchical spatiotemporal framework underlying the analysis, and then display the results of fitting various space–time formulations to the malaria data with the different model selection metrics. The Bayesian model selection process assessed the sensitivity of different specifications to obtain the optimal models for the analyses. We also evaluated the malaria model performance retrospectively based on the 2015–2021 national surveillance data. To assess whether malaria could be eliminated by 2024 per Thailand’s National Malaria Elimination Strategy, 2017–2026, we used the best-fitted model to project the estimated cases for 2022–2028.

## Methods

### Malaria surveillance data

Previous literature suggests that a well-established and integrated surveillance system could serve as an instructive infrastructure for planning disease control and elimination strategies^1,2,8^. The dramatic reduction of malaria incidence in Thailand was partly because of an effective electronic-based malaria surveillance system which included inputs and actions from all related stakeholders at the national and local levels^2^. The Division of Vector-borne Diseases (DVBD), in collaboration with the Center of Excellence in Biomedical and Health Informatics (BIOPHICS) of the Faculty of Tropical Medicine, Mahidol University, has developed a web-based Malaria Information System^8^, which has been modified and scaled-up nationwide. The system collects assorted data, including incident cases, diagnostics, locations, travel histories, vector characteristics and behavior in endemic villages and prevention and control activities such as insecticide-treated net distributions and indoor spraying of houses. The system’s functionalities include analyses and automated presentations of information and reports in table, graph and map formats. The system also generates granular information on malaria cases and affected villages (foci) which can be used for planning and monitoring malaria elimination activities^2,8^. This study analyzed 2015–2021 data from this malaria information system. The analyses were conducted using secondary data. This study was waived for informed consent of participants and approved by the Institutional Review Board of the Faculty of Tropical Medicine, Mahidol University (MUTM EXMPT 2022-002). All methods were carried out in accordance with relevant guidelines and regulations.

### Bayesian spatiotemporal modeling for provincial malaria incidence

To estimate malaria incidence, classical approaches do not consider the spatial dependence among the areas^9^. We applied Bayesian spatiotemporal modeling that considers both spatial and temporal dependence among space and time dimensions. A Bayesian disease mapping model consists of three components: likelihood (the distribution of the data given the parameters), the process model (a description of underlying spatiotemporal pattern) and the parameter model (the prior knowledge of the parameters to be estimated)^10^. We modeled the malaria data from the passive surveillance database consisting of aggregate case counts in each province. The Negative Binomial (NB) base distribution with an overdispersion parameter was considered to capture the variability in the likelihood. This gives rise to

1 $$y_{it} \sim Negtive\;Binomial(n_{it} \theta_{it} ,\phi )$$

where $y_{it}$ is the number of new malaria cases in province *i* and year *t*. Although the Poisson likelihood has been widely applied as a standard practice in Bayesian disease mapping^11–14^, assuming a negative binomial model was appealing because of overdispersion, i.e. its variance exceeds the mean, is evident relatively to the Poisson^15^. Particularly, $E(y_{it} ) = \mu_{it} = n_{it} \theta_{it}$ is the conditional mean of the Negative Binomial base distribution and $Var(y_{it} ) = \mu_{it} (1 + \mu_{it} /\phi )$ where $n_{it}$ is the offset defined as the population at risk for each location and time. The quantity $1/\phi$ is an overdispersion parameter and as $1/\phi \to 0$, the negative binomial converges to a Poisson distribution corresponding to no overdispersion.

The process and parameter models were formulated to describe the underlying structure and associated parameters linked to the mean incidence. Many processes and models allowing for space and time variation of disease incidence have been proposed in the literature (see examples^12,16–18^). A log-linear model is usually applied in standard Bayesian disease mapping to link with the linear predictor as $\log (\mu_{it} ) = \log (n_{it} ) + \log (\theta_{it} )$. We included both structured and unstructured random effects in the log-linear predictor to counter unmeasured confounders. The unstructured random effect, $v_{i}$, is often modeled by a Normal prior distribution with zero mean and variance $\sigma_{v}^{2}$. The spatially structured effect, $u_{i}$, is usually specified as the intrinsic conditional autoregressive (ICAR) model developed by Besag et al.^19^. This prior has a conditional form of $u_{i} |{\varvec{u}}_{ - i} \sim N\left( {\overline{u}_{{\Omega_{i} }} ,{{\sigma_{u}^{2} } \mathord{\left/ {\vphantom {{\sigma_{u}^{2} } {n_{{\delta_{i} }} }}} \right. \kern-0pt} {n_{{\delta_{i} }} }}} \right)$ where the vector ${\varvec{u}}_{ - i}$ represents the correlated effect of all except the *i*th province. $\Omega_{i}$, $n_{{\delta_{i} }}$ and $\overline{u}_{{\delta_{i} }}$ are a set of the first-order spatial neighbors, cardinality and the average of the neighborhood of the *i*th province respectively.$\sigma_{u}^{2}$ is the variance of spatial component. Thus, we have the spatial component of the model as $\log (\theta_{it} ) = \beta_{0} + v_{i} + u_{i}$ where $\beta_{0}$ is the overall intercept.

To extend the process and parameter models to both space and time dimensions, Bernardinelli et al*.* proposed a Bayesian model with parametric time trends^20^. A main linear time effect and differential time trend for each location were added to the spatial components. The linear predictor is thus written as $\log (\theta_{it} ) = \beta_{0} + v_{i} + u_{i} + (\beta_{1} + \varphi_{i} )t$. The parameter $\beta_{1}$ represents an overall linear time trend whereas $\varphi_{i}$ captures the interaction between the linear time trend and the location effect *v*_i_ and *u*_i_, respectively. This formulation shares similarities with the random slope modeling. We imposed a linearity constraint on the differential temporal trend $\varphi_{i}$, nonetheless it is possible to remove it using a dynamic nonparametric formulation for the linear predictor as.

2 $$\log (\theta_{it} ) = \beta_{0} + v_{i} + u_{i} + (\beta_{1} + \varphi_{i} )t + \phi_{t} + \delta_{it} .$$

The nonparametric term $\phi_{t}$ represents the temporally structured effect which can be modeled as a random walk prior of order 1 or 2, or a Gaussian exchangeable prior. The specification of the prior on $\delta_{it}$ depends on the spatial and temporal main effects, which are assumed to interact. Four types of interactions are proposed^21^, and each interaction type can be interpreted in a different way creating a large number of possible models for the provincial malaria incidence. More details on model specification, hotspot analysis, and computation used in this study can be found in the “Supplementary Document”.

### Model sensitivity analysis and selection criteria

To assess the model sensitivity and performance of different specifications in the previous section, we applied various evaluation metrics to measure model outcomes. Five metrics evaluated the performance of developed models: deviance information criterion (DIC), Watanabe-Akaike information criterion (WAIC), bias, root mean squared error (RMSE), and Spearman’s correlation coefficient. Forms of model assessment involve measuring the goodness-of-fit (GOF) to evaluate whether the particular data in space and time provide an adequate fit to the model. The deviance information criterion (DIC)^22^ has been widely used for overall model fit in Bayesian settings generalized from the Akaike information criterion (AIC) in the Frequentist framework. Another is the Watanabe-Akaike information criterion (WAIC)^23^ considered an improved DIC version. WAIC is fully Bayesian, applying the entire use of the posterior distribution, and unlike DIC, it is robust against different parametrizations and is valid for singular models^24^. To investigate the estimation uncertainty, we calculated the RMSE, as the squared root of the average squared deviation between the observed and estimated means across the provinces and study time periods*.* We also calculated the Spearman’s correlation coefficient between the observed and estimated malaria cases. Since the malaria incidence data in the elimination period are not normally distributed^13,14^, Spearman’s correlation coefficient was the most appropriate. Finally, we also employed the conditional predictive ordinate (CPO), a model assessment criterion with cross-validation using posterior sampling, computed as $CPO_{it} = P(y_{it} \user2{|y}_{ - it} )$^17^. For each observation, *CPO*_*it*_ was calculated by fitting the model with all the data except for the observation *y*_*it*_. The metric was then computed as $- \sum\nolimits_{t} {\sum\nolimits_{i}^{{}} {\log (CPO_{it} )} }$ and a smaller value of this quantity indicates a better fit of the model to the data.

### Model selection and prediction

In this study, we analyzed the surveillance data from 77 Thai provinces and selected hotspots of high-risk provinces along its three international borders: northwest (Thailand–Myanmar), northeast (Thailand–Cambodia) and south (Thailand–Malaysia). As the epidemiology of the two dominant malaria species in Thailand were different and elimination efforts would require different strategies, we performed separate model fits for *P. falciparum* and *P. vivax*.

Based on the identified best-fitted models for the observed surveillance cases per 100,000 population at the provincial level in 2015–2021, we predicted the number of cases per 100,000 population in 2022–2028. The predicted malaria cases were computed from the optimal models derived from the model selection process for each species.

### Ethics approval

This study was waived for informed consent of participants and approved by the Institutional Review Board of the Faculty of Tropical Medicine, Mahidol University (MUTM EXMPT 2022-002). All methods were carried out in accordance with relevant guidelines and regulations.

## Results

### Reported malaria cases during 2015–2021

**Figure 1:**
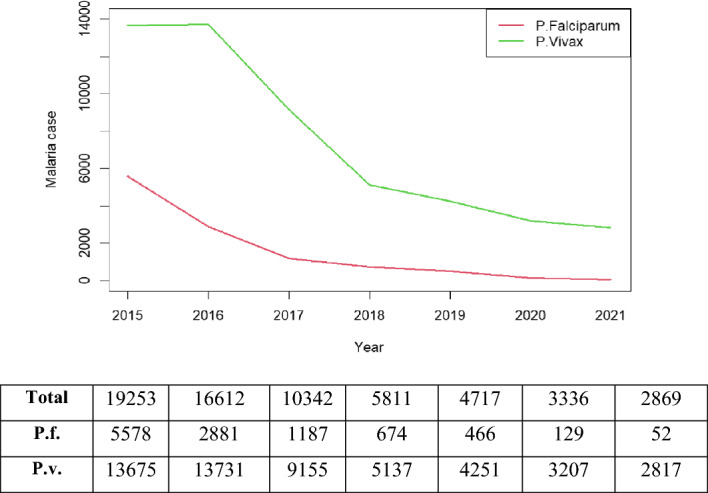
Annual number of *P. falciparum* and *P. vivax* malaria cases from the national passive surveillance database during 2015–2021*.* Note: Total = Pf + Pv. (Figure prepared using RStudio 2022.02.3 + 492 "Prairie Trillium", www.r-project.org/).

Thailand experienced a decrease in malaria cases from 2015 to 2021. As shown in Fig. 1, the total number of cases over the seven years was 10,967 cases for *P. falciparum* and 51,973 for *P. vivax.* The number of *P. falciparum* cases dropped from 5578 in 2015 to 52 in 2021. The number of *P. vivax* cases dropped from 13,675 in 2015 to 2817 in 2021.

### Spatiotemporal model specification

We constructed different spatiotemporal model formulations (see the Supplementary Document S1–S3 and S6 for more details) with evaluation measures. As random effects can be used in different ways, we thus included them in our models to account for additional space–time variation in either structured or unstructured terms. We proposed model variants with different forms of random effects. For spatial random effects, we applied an independent zero-mean Gaussian prior as the unstructured terms in the model, and an Intrinsic Conditional Autoregressive (ICAR), i.e. Besag and BYM, combined with the unstructured in other models. For the temporal dimension, the structured part was provided as the fixed effect of linear time (under log link) and the unstructured was modeled by Gaussian exchangeable prior. Thus, the model specifications covered various forms of space–time variation with both structured and unstructured terms.

Table 1 displays the results of all 18 model forms that we explored. In models 1–6, only the random intercept terms were included in the analysis. For the first two models, we captured the temporal variation in the parametric linear form with a common coefficient. The spatial random effect with the independent zero-mean Gaussian prior was only included in model 1, while the random intercept was implemented using both the unstructured and Besag (BYM) prior in model 2. Models 3–4 were similar to models 1–2, but the nonparametric dynamic trend was added to capture the temporal variation in addition to the parametric term. The models allowed for interaction between provinces and time periods, was added in models 5 and 6. Models 7–12 were specified as random slope modeling with a zero-mean Gaussian exchangeable prior on the temporal coefficient. Models 13–18 were assumed to have a BYM prior for each provincial slope while accounting for spatial structure on the temporal coefficient.

**Table 1:** Spatiotemporal model selection for total malaria cases reported within the national malaria surveillance system under different evaluation measures.

Model specification		*P. falciparum*					*P. vivax*				
		DIC	WAIC	CPO	RMSE	Correlation	DIC	WAIC	CPO	RMSE	Correlation
1	$\beta_{0} + v_{i} + \beta_{1} t$	1952.04	1954.56	979.7706	1319.66	0.833	2937.93	2938.49	1481.57	5097.74	0.871
2	$\beta_{0} + v_{i} + u_{i} + \beta_{1} t$	1944.26	1948.84	976.6073	1389.93	0.833	2936.44	2937.27	1480.909	5134.02	0.871
3	$\beta_{0} + v_{i} + \beta_{1} t + \phi_{t}$	1951.87	1954.38	980.7353	1320.14	0.833	2937.96	2938.42	1480.313	5098.28	0.871
4	$\beta_{0} + v_{i} + u_{i} + \beta_{1} t + \phi_{t}$	1944.45	1948.23	975.7881	1388.12	0.834	2932.67	2933.84	1479.207	5582.71	0.870
5	$\beta_{0} + v_{i} + \beta_{1} t + \phi_{t} + \delta_{it}$	1950.35	1947.86	980.549	1219.11	0.838	2937.82	2937.01	1479.643	5091.68	0.871
6	$\beta_{0} + v_{i} + u_{i} + \beta_{1} t + \phi_{t} + \delta_{it}$	3265.29	3264.90	975.8496	14,786,840	0.047	2933.12	2934.33	1479.322	5582.75	0.870
7	$\beta_{0} + v_{i} + \beta_{1i} t$	1928.09	1917.69	1269.74	1105.02	0.857	2866.06	2861.71	1456.68	4924.84	0.896
8	$\beta_{0} + v_{i} + u_{i} + \beta_{1i} t$	3265.07	3265.04	1271.623	14,778,320	0.042	2864.84	2860.31	1482.521	4981.46	0.896
9	$\beta_{0} + v_{i} + \beta_{1i} t + \phi_{t}$	1893.01	1895.27	958.7819	1106.91	0.855	2839.20	2836.38	1437.938	5122.64	0.897
10	$\beta_{0} + v_{i} + u_{i} + \beta_{1i} t + \phi_{t}$	1889.57	1889.85	956.4324	1250.99	0.856	**2839.23**	**2835.83**	**1437.876**	**5121.92**	**0.897**
11	$\beta_{0} + v_{i} + \beta_{1i} t + \phi_{t} + \delta_{it}$	3265.34	3265.06	16,985	14,692,530	0.051	4457.59	4457.41	2085.997	11,515,190	0.064
12	$\beta_{0} + v_{i} + u_{i} + \beta_{1i} t + \phi_{t} + \delta_{it}$	3265.21	3265.00	36,325.52	14,793,130	0.042	2865.98	2861.49	1479.603	4940.03	0.896
13	$\beta_{0} + v_{i} + \beta s_{1i} t$	3265.18	3265.14	1293.974	–	–	4457.98	4457.53	1489.904	–	–
14	$\beta_{0} + v_{i} + u_{i} + \beta s_{1i} t$	1923.90	1915.88	1236.543	1290.56	0.857	4453.43	4457.44	1476.241	11,668,590	0.056
15	$\beta_{0} + v_{i} + \beta s_{1i} t + \phi_{t}$	1893.35	1896.39	961.5975	1329.73	0.850	4420.25	4423.98	1447.454	6,955,985	-0.017
16	$\beta_{0} + v_{i} + u_{i} + \beta s_{1i} t + \phi_{t}$	1883.37	1886.50	953.3092	1512.20	0.852	67,920.58	103,280.35	1467.131	8.31E + 91	-0.352
17	$\beta_{0} + v_{i} + \beta s_{1i} t + \phi_{t} + \delta_{it}$	**1764.73**	**1747.49**	**959.8387**	**131.35**	**0.888**	4457.60	4457.29	1886.988	11,619,540	0.060
18	$\beta_{0} + v_{i} + u_{i} + \beta s_{1i} t + \phi_{t} + \delta_{it}$	3265.37	3265.10	1394.047	14,871,990	0.038	4457.57	4457.33	1483.143	11,674,360	0.057

Significant values are in [bold].

Various evaluation metrics were computed to assess the performance of different models for each malaria species. Based on these metrics, we selected the models that had the best overall performance. Table 1 shows the results of our model selection for total malaria cases. model 17, which contained the unstructured spatial random effect, temporal parametric with spatial random slope, nonparametric temporal and their interaction terms, had the best performance. Though models 16 and 17 had the lowest CPO values, with model 16 having a slightly better CPO value, considering other model criteria, model 17 had the best performance based on various model fit indicators and was selected as the best overall fit for total *P. Falciparum* malaria cases. For total *P. vivax* malaria cases, models 9 and 10 performed similarly. However, model 10, which included the BYM prior, temporal parametric with non-spatial random effect and nonparametric temporal terms, yielded a better CPO value with a better value of WAIC. Therefore, we selected model 10 to interpret the results for total *P. vivax* malaria cases. In Table 2, we presented the spatiotemporal model selection results for indigenous malaria cases under different evaluation measures. Model 9 was chosen to interpret the results for both spicies based on the best overall performance across these measures. (Note blank cells in Tables 1 and 2 were values that could not be computed. This might be because the model specifications were not suitable for the data used in this project.)

**Table 2:** Spatiotemporal model selection for indigenous malaria cases who locally contracted with no evidence of importation or link to the imported case under different evaluation measures.

Model specification		*P. falciparum*					*P. vivax*				
		DIC	WAIC	CPO	RMSE	Correlation	DIC	WAIC	CPO	RMSE	Correlation
1	$\beta_{0} + v_{i} + \beta_{1} t$	1467.02	1466.48	577.4443	11,804.12	0.220	1720.01	1704.76	912.3844	10,413.75	0.770
2	$\beta_{0} + v_{i} + u_{i} + \beta_{1} t$	1812.61	1811.82	1170.011	2.46E + 07	0.065	2595.16	2594.97	856.6552	24,278,245	0.065
3	$\beta_{0} + v_{i} + \beta_{1} t + \phi_{t}$	1812.35	1811.91	575.8684	2.46E + 07	0.064	1719.35	1704.58	862.1396	10,414.83	0.770
4	$\beta_{0} + v_{i} + u_{i} + \beta_{1} t + \phi_{t}$	1254.99	3012.09	1211.504	4.00E + 22	0.675	1703.08	1695.45	857.2879	10,440.62	0.770
5	$\beta_{0} + v_{i} + \beta_{1} t + \phi_{t} + \delta_{it}$	1486.41	1488.43	42,276.31	11,795.26	0.192	2595.00	2594.99	861.7181	24,250,633	0.064
6	$\beta_{0} + v_{i} + u_{i} + \beta_{1} t + \phi_{t} + \delta_{it}$	1254.33	2973.00	1335.785	7.68E + 22	0.676	2595.17	2594.93	11,561.31	24,278,489	0.065
7	$\beta_{0} + v_{i} + \beta_{1i} t$	1812.30	1812.12	696.9095	2.46E + 07	0.064	2595.21	2594.89	913.3691	24,264,984	0.064
8	$\beta_{0} + v_{i} + u_{i} + \beta_{1i} t$	1812.52	1812.01	1787.722	2.46E + 07	0.0653	1710.08	1702.16	863.5288	10,711.53	0.777
9	$\beta_{0} + v_{i} + \beta_{1i} t + \phi_{t}$	**1071.32**	**1045.07**	**565.648**	**11,573.53**	**0.687**	**1670.22**	**1656.51**	**841.1158**	**10,338.08**	**0.791**
10	$\beta_{0} + v_{i} + u_{i} + \beta_{1i} t + \phi_{t}$	1242.40	5288.63	2191.439	1.37E + 23	0.680	2595.12	2594.94	956.2627	24,249,853	0.064
11	$\beta_{0} + v_{i} + \beta_{1i} t + \phi_{t} + \delta_{it}$	1812.14	1812.46	706.4263	2.46E + 07	0.064	1631.72	1618.39	913.7699	10,167.02	0.701
12	$\beta_{0} + v_{i} + u_{i} + \beta_{1i} t + \phi_{t} + \delta_{it}$	1812.15	1812.49	1840.642	2.46E + 07	0.065	2595.08	2595.08	933.7631	24,286,765	0.064
13	$\beta_{0} + v_{i} + \beta s_{1i} t$	1812.14	1812.02	6686.582	–	–	2595.37	2594.91	882.6302	–	–
14	$\beta_{0} + v_{i} + u_{i} + \beta s_{1i} t$	1812.26	1812.33	5852.4	2.47E + 07	0.064	2595.18	2595.16	869.6824	24,359,758	0.064
15	$\beta_{0} + v_{i} + \beta s_{1i} t + \phi_{t}$	1812.45	1811.95	4948.827	2.47E + 07	0.063	2595.12	2594.89	1373.024	24,292,479	0.063
16	$\beta_{0} + v_{i} + u_{i} + \beta s_{1i} t + \phi_{t}$	–	1004.89	5546.253	–	0.604	2595.16	2595.02	876.4885	24,318,188	0.064
17	$\beta_{0} + v_{i} + \beta s_{1i} t + \phi_{t} + \delta_{it}$	1812.07	1811.88	6722.076	2.47E + 07	0.063	2595.08	2595.22	86,827.36	24,330,567	0.063
18	$\beta_{0} + v_{i} + u_{i} + \beta s_{1i} t + \phi_{t} + \delta_{it}$	–	1046.85	5596.644	5.54E + 112	0.612	2595.12	2595.11	870.0114	24,358,246	0.064

Significant values are in [bold].

### Malaria incidence at provincial level and hotspots

**Figure 2:**
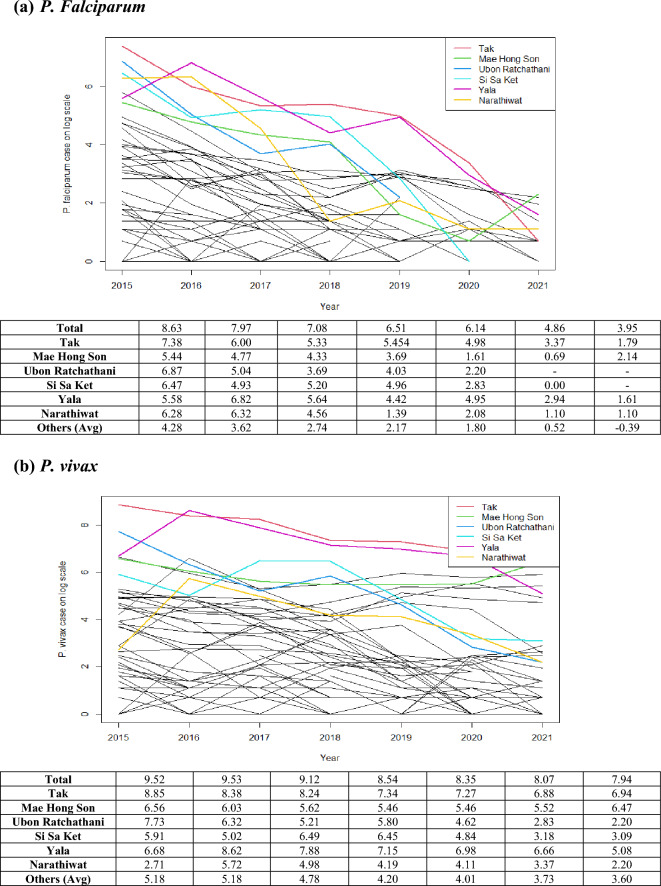
Annual number of cases (log scale) in 77 Thai provinces and 6 Hotspot Provinces, during 2015–2021: (**a**) *P. falciparum* cases and (**b**) *P. vivax* cases. *Note: log(1)* = *0 and log(0)* = *− In.* (Figure prepared using RStudio 2022.02.3 + 492 "Prairie Trillium", www.r-project.org/).

Table 3 presents malaria incidence per 100,000 population for both species in hotspots of Thai provinces during 2015–2021, while plots in Fig. 2 showed malaria cases on the natural logarithm scale of all provinces and hotspots. The trend of malaria cases appeared to be approximately linear under the log scale during the study period. A hotspot could be defined as any isolated location or geographically-bounded region that displays an excess of disease risk or incidence in a particular time. Since we examined the data over a 7-year period, we performed the analysis to find persistent hotspots over the study period based on the population at risk in each province averaged over the study period. We determined hotspots using a space–time anomaly detection approach by identifying exceedance probability from the number of estimates in the posterior sample which exceed a pre-specified threshold (see Supplement Document S2 for hotspot analysis details).

**Table 3:** Annual incidence of malaria cases per 100,000 population in 77 Thai provinces and 6 hotspot provinces during 2015–2021. Note that the other average is the case average over other provinces excluding the 6 hotspots.

	2015	2016	2017	2018	2019	2020	2021
	*P. falciparum*						
Total	9.52	9.53	9.12	8.54	8.35	8.07	7.94
Tak	8.85	8.38	8.24	7.34	7.27	6.88	6.94
Mae Hong Son	6.56	6.03	5.62	5.46	5.46	5.52	6.47
Ubon Ratchatani	7.73	6.32	5.21	5.83	4.63	2.83	2.20
Si Sa Ket	5.91	5.02	6.49	6.45	4.84	3.18	3.09
Yala	6.68	8.62	7.88	7.15	6.98	6.66	5.08
Narathiwat	2.71	5.72	4.98	4.19	4.11	3.37	2.20
Other average	5.18	5.18	4.78	4.20	4.01	3.73	3.60
	*P.Vivax*						
Total	8.63	7.97	7.08	6.51	6.14	4.86	3.95
Tak	7.38	6.00	5.33	5.45	4.98	3.37	1.79
Mae Hong Son	5.44	4.77	4.33	3.69	1.61	0.69	2.14
Ubon Ratchatani	6.87	5.04	3.69	4.03	2.20	-Inf	-Inf
Si Sa Ket	6.47	4.93	5.20	4.96	2.83	0.00	-Inf
Yala	5.58	6.82	5.64	4.42	4.95	2.94	1.61
Narathiwat	6.28	6.32	4.56	1.39	2.08	1.10	1.10
Other average	4.28	3.62	2.74	2.17	1.80	0.52	-0.39

**Figure 3:**
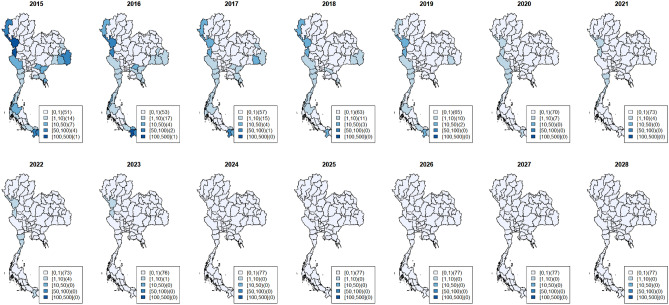
Maps of observed surveillance (2015–2021) and prediction (2022–2028) of total *P. Falciparum* cases per 100,000 population at provincial level. (Figure prepared using RStudio 2022.02.3 + 492 "Prairie Trillium", www.r-project.org/).

**Figure 4:**
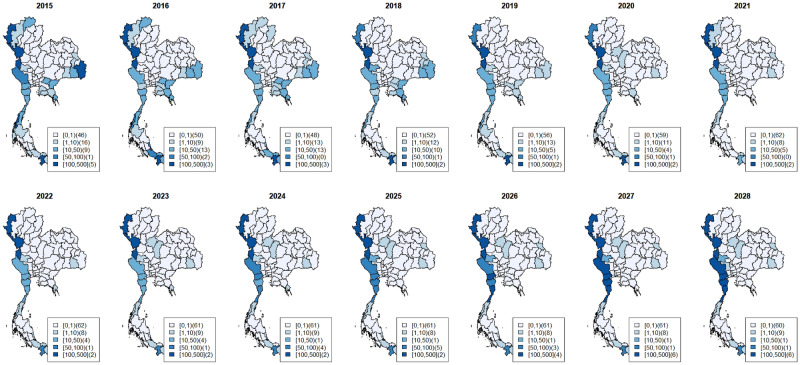
Maps of observed surveillance (2015–2021) and prediction (2022–2028) of total *P. Vivax* cases per 100,000 population at provincial level. (Figure prepared using RStudio 2022.02.3 + 492 "Prairie Trillium", www.r-project.org/).

Since 2015, there have been only a few *P. falciparum* cases per 100,000 population, especially in the central, north, and northeastern regions, except for the border areas with neighboring countries. The hotspot analysis showed six high-risk provinces, two in the northwest (Tak and Mae Hong Son), the northeast (Ubon Ratchathani and Si Sa Ket) and the south (Yala and Narathiwat). The overall trend has been monotonic decreasing (Fig. 2) as in all provinces. However, in the west, *P. falciparum* cases showed a slower decline. Overall the number of *P. vivax* cases was gradually decline over the years. The hotspot analysis showed three persistent high-risk provinces in the northwest (Mae Hong Son and Tak) and one in the south (Yala); among the six hotspot provinces, slower declining trends in these three provinces were observed with higher incidences in 2021. The spatial pattern of vivax cases was similar to falciparum but more prevalent, particularly along the borders (Figs. 3, 4). Since 2020, the high-risk areas have decreased, mostly in the west and south among the hotspot provinces.

### Observed incidences (2015–2021) and predicted incidences (2022–2028)

Figures 3 and 4 depicted the maps of *P. falciparum* and *P. vivax* observed surveillance cases (2015–2021, top row) and prediction (2022–2028, bottom row) per 100,000 population at the provincial level (Table 1). Most provinces, especially in the central, east and northeast, displayed a decreasing trend for both species.

As shown in Fig. 3, the observed surveillance data showed that since 2020, *P. falciparum* cases had dropped to less than 1 case per 100,000 population in all provinces except in the west. The predicted *P. falciparum* cases will continue to decrease all over the country. The last region with < 1 case per 100,000 is the west, and the entire country will achieve < 1 case per 100,000 in 2024. From 2015 to 2021, the number of provinces with zero *P. falciparum* cases increased from 51 provinces (2015) to 73 provinces (2021). Based on our selected model, from 2024 onward, all 77 provinces would have zero *P. falciparum* cases. We also projected estimates for 2022–2024 in the six hotspots and, similarly, in all 77 provinces, which would have zero *P. falciparum* cases from 2024 onward (see Supplementary Document S4 presenting the plots of *P. falciparum* surveillance case estimates with 95% credible band of hotspot provinces).

*P. vivax* cases have been more prevalent in provinces (Fig. 4). The overall trend shows a decline with scattered increases in some regions. Hotspots in the west had the highest number of cases during 2015–2021 and will persist during the projected period. The number of provinces with zero *P. vivax* cases increased from 46 in 2015 to 62 in 2021. Our selected model predicts that 61 provinces will achieve zero *P. vivax* cases during 2023–2028. Our estimates for 2022–2024 in the 6 hotspots indicated unstable trends—a slow decrease in some hotspots but an increase in others with high prediction uncertainty; for example, a hotspot in the south (Yala Province) and the west could potentially have an increase with large uncertainty in the future (see Supplementary Document S4 and S5 showing plots of *P. vivax* surveillance case estimates with 95% credible band of hotspot provinces).

**Figure 5:**
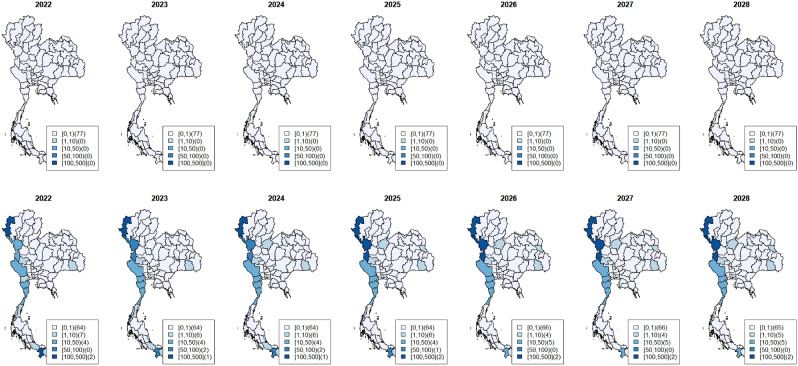
Maps of prediction (2022–2028) of indigenous *P. falciparum* cases (top row) and *P. vivax* cases (bottom row) per 100,000 population at provincial level. (Figure prepared using RStudio 2022.02.3 + 492 "Prairie Trillium", www.r-project.org/).

Figure 5 depicts the maps of predicted incidence per 100,000 population at the provincial level in 2022–2028 for only indigenous cases of *P. falciparum* (top row) and *P. vivax* (bottom row). Similar results were predicted for all malaria cases. Most provinces, especially in the central, east and northeast, had a decreasing trend in both species. However, based on the selected model, all 77 provinces would have zero indigenous *P. falciparum* cases from 2022 onward, and 65 provinces would have zero indigenous *P. vivax* cases during 2023–2028.

## Discussion

In keeping with WHO’s “zero malaria” campaign, Thailand has pushed forward to eliminate malaria by 2024. However, as in the Greater Mekong subregion (GMS), Thailand has been concerned about reaching this important goal due to the COVID-19 pandemic^25–27^. This study is the first to develop Bayesian hierarchical spatiotemporal models utilizing the Thai malaria surveillance database that included data during the pandemic (2019–2021) to predict malaria incidence for the two dominant species, *P. falciparum* and *P. vivax,* for 2022–2028. In the model development process, we applied Bayesian model selection techniques to choose the optimal models to retrospectively analyze observed surveillance malaria data and estimate future cases for appropriate disease planning. These methods offer a very flexible modeling approach which can accommodate and potentially select between a wide range of space–time linear predictors^7,28–30^.

We constructed hierarchical spatiotemporal models that confirmed a drastic decrease in both *P. falciparum* and *P. vivax* cases nationwide. A potential contributing factor could be the “1–3–7” surveillance strategy adopted by the National Malaria Elimination Strategy 2017–2026. The strategy includes case reporting within 24 h, case investigation and classification within 3 days, and foci investigation and response within 7 days. The strategy has proved effective in China^2,31^, reaching zero indigenous malaria cases in 2018. However, recent reports suggest China’s success was aided by the application of innovative genetics-based approaches^32^, which included surveying malaria parasite populations and drug resistance. To achieve zero malaria by 2024, Thailand may need to implement similar approaches at the local health facility level.

Despite the observed decrease in nationwide malaria cases, the developed models also reflected high spatial heterogeneity in malaria incidence across the country. Our spatiotemporal and hotspot analysis revealed persistent malaria transmission along the border areas with Myanmar, Cambodia and Malaysia. As noted in the President’s Malaria Initiative plan, the remaining active foci in Thailand are clustered in three border areas because high population mobility is associated with the importation of malaria parasites and drug resistance in the west (Myanmar) and the east (Cambodia), whereas civil unrest has disrupted service delivery in the south (Malaysia)^33^. Migration and cross-border population mobility may have influenced the disease incidence^25,34^. A recent study on the Thailand-Myanmar border found a higher probability of villages becoming a malaria infection hotspot where the border could be easily crossed^35^. Differing occupational activities and access to malaria diagnosis and treatment along the border between neighboring countries^1,4,35^ as well as mobility of asymptomatic individuals, re-introducing infection into communities, and drug-resistant populations could prevent achieving zero malaria^35–37^. Thus, the WHO’s operational framework for cross-border collaboration to secure a malaria-free South-East Asia Region (2018) suggested member states prevent and/or reduce transmission and disease burden with special emphasis on minimizing the risk of importation of malaria cases^38^

To achieve zero malaria by 2024, Thailand's medium-term goal is zero malaria transmission in 95% of all districts by 2021^1^. Our models of provincial levels have shown that 73 of 77 provinces had zero *P. falciparum* cases in 2021. Further, our models estimate that all 77 provinces would have zero *P. falciparum* cases after 2024. However, for *P. vivax*, our models predict 62 provinces with zero *P. vivax* cases with no increase by 2028. WHO certification of malaria elimination requires applicant countries to provide evidence that the local malaria transmission has been fully interrupted, resulting in zero indigenous malaria cases for at least 36 months^39^. Our models predicted zero *P. falciparum* cases by 2024, but not zero *P. vivax* cases.

As discussed in previous reports, *P. vivax* malaria control efforts have been less successful than for *P. falciparum*^40–42^. The prediction model suggested that P. falciparum could be eliminated earlier than P. vivax; this may, in part, be due to the intensive effort of the national malaria control program to eliminate resistance to artemisinin combination therapies with early diagnosis and efficacious drug administratiion in the reported problematic areas. Although prompt diagnosis and treatment of symptomatic patients are effective measures to prevent severe disease and reduce transmission, their success may be undermined by asymptomatic individuals and hidden reservoirs not captured in the malaria database^41^. *P. vivax-*infected individuals may have very low asexual parasite densities, mixed infections with *P. falciparum*, or undetectable hypnozoites^40^. Eliminating *P. vivax* malaria would require different approaches from those seen with *P. falciparum*, such as targeted vector control and availability of detection services (microscopic tests, genetic-based technique, or bivalent rapid diagnostic tests)^40^. One such initiative is mass drug administration (MDA), wherein the antimalarial treatment is offered to communities regardless of an individual’s malaria infection status. For the radical cure of *P. vivax,* the MDA approach must be tailored to specific settings, including targeting high-risk populations and isolated communities^27^. A study in Brazil confirms that the standard treatment drug (i.e., primaquine) when administered at the right dose for a sufficient time, is effective in preventing the recurrence of *P. vivax* malaria.^42^. A cross-sectional mixed-methods study on MDA to reduce vivax malaria in a northern Myanmar township suggested that most respondents agreed to participate in the proposed mass treatment campaign and that the community engagement process increased community acceptance^43^. Another approach to accelerate *P. vivax* elimination is a safe and protective vaccine, but it is unlikely to be available in the near future^44^. For Thailand to reach zero *P.vivax*, there should be *P. vivax*-specific control and elimination plans with specific indicators on program coverage and disease incidence.

There are some limitations in model development and prediction of malaria incidence. Bayesian models are traditionally fitted through Markov Chain Monte Carlo sampling (MCMC). Due to the nature of the hierarchical models and random effects, the convergence of MCMC can be very slow. Integrated Nested Laplace Approximation has recently been developed as an alternative method to fit Bayesian hierarchical. However, one comparison shows thar INLA is equivalent to MCMC for parameter estimation in disease mapping studies. In addition, the overall computational burden of INLA was much lower^45^ We then applied INLA in this project; a comparison among posterior approximation methods will be further investigated. The hierarchical models constructed in this study included various forms of both space and time random effects, which accounted for some unmeasured confounding effects^12,29^. We did not consider vector and environmental factors that may influence the prediction model. We encountered a computing challenge when performing the model selection, specifically, the strength of incidence trend present in the data. If there was insufficient temporal evidence in the data, the model might not be precisely identified. The prediction model fitted well for *P. falciparum* hotspots. However, for *P. vivax,* our estimation failed to perform well with greater uncertainty. The wide credible band of *P. vivax* estimates might be because of spatial and temporal data fluctuations, particularly for *P. vivax* reported cases in malaria-endemic areas. Despite these limitations, we developed a flexible spatiotemporal modeling platform that can be further modified when more resources and data are available. We believe our investigation is beneficial to understanding the spatiotemporal malaria patterns to support decisions in malaria control and elimination activities in Thailand.

## Conclusions

The estimation of malaria incidence was determined using Bayesian hierarchical spatiotemporal models. Hotspots analysis based on the best-fitted models for *P. falciparum* and *P. vivax* identified several Thai provinces that persistently carry a relatively high malaria burden compared to other provinces. To project the possibility of meeting WHO’s criteria of “zero malaria” country by 2024, the models can predict malaria incidence for 2022–2028. To receive WHO’s malaria-free certification is to have investigation forms and maps on focus management and response to demonstrate the effectiveness of activities to interrupt transmission in the last foci. The spatiotemporal maps based on the models revealed different predicted estimates between both species. The model for *P. falciparum* suggested that zero *P. falciparum* cases might be possible by 2024, in contrast to the model for *P. vivax,* wherein zero *P. vivax* cases might not be reached.

The challenges to eliminating *P. vivax* are well recognized. It likely requires timely radical cure. Innovative approaches and best practices to improve case detection and treatment rates are essential. Further, when evaluating malaria programs in low transmission areas, surveillance should be a case-based system that can identify high-risk groups in subnational areas so that all related stakeholders can timely review and take immediate action^46^. For Thailand, to monitor activities in moving towards zero malaria, effective methods for area stratification or classification according to transmission intensity are required. Innovative approaches in the *P. vivax*-specific control and elimination plans must be implemented to reach zero *P. vivax* and consequently declare Thailand as a malaria-free country.

## Supplementary Information

Supplementary Information.

## Data Availability

The data used in this study were obtained from the Division of Vector Borne Diseases, Department of Disease Control, Thai Ministry of Public Health, but restrictions apply to the availability of these data, which were used with permission for the current study, and are therefore not publicly available. However, data may be available from the authors upon a reasonable request and with permission of the Division of Vector Borne Diseases.
