# Supplementary material for: Projecting malaria elimination in Thailand using Bayesian hierarchical spatiotemporal models
Source: Sci Rep. 2023 May 13;13:7799. doi: 10.1038/s41598-023-35007-9 (PMC10182757; doi:10.1038/s41598-023-35007-9)
Supplement: Supplementary file 1 — Supplementary Information. [file 41598_2023_35007_MOESM1_ESM.docx]

**Supplementary document**

**S1: Bayesian spatiotemporal model specification for provincial malaria incidence**

There are a large number of possible models for spatiotemporal malaria incidence data. In this study we limited the model specification to those sensible for our modeling purpose. All model forms used in this study was shown in table S1 below. In models 1-6, only the random intercept terms were included in the analysis. The first two model the temporal variation was only captured in the parametric linear form with common coefficient$\beta_{0}$. The spatial random effect with the independent zero-mean Gaussian prior was only included in model 1 while the random intercept was implemented using both the unstructured and Besag (BYM) prior in model 2 [1]. Models 3-4 were similar to models 1-2 but the nonparametric dynamic trend, , was added to capture the temporal variation in addition to the parametric term. was specified by means of a Gaussian exchangeable prior. Next, to expand the models to allow for an interaction between provinces and time periods,$\delta_{it}$was added in models 5 and 6.

| Model | Linear predictor specification |
| --- | --- |
| 1 | $\beta_{0}+v_{i}+\beta_{1}t$ |
| 2 | $\beta_{0}+v_{i}+u_{i}+\beta_{1}t$ |
| 3 |  |
| 4 |  |
| 5 |  |
| 6 |  |
| 7 | $\beta_{0}+v_{i}+\beta_{1i}t$ |
| 8 | $\beta_{0}+v_{i}+u_{i}+\beta_{1i}t$ |
| 9 |  |
| 10 |  |
| 11 |  |
| 12 |  |
| 13 | $\beta_{0}+v_{i}+\beta s_{1i}t$ |
| 14 | $\beta_{0}+v_{i}+u_{i}+\beta s_{1i}t$ |
| 15 |  |
| 16 |  |
| 17 |  |
| 18 |  |

**Table S1. Model specifications with different forms of space-time random effects.**

There are four types of interactions are proposed in [2] and each interaction type can be interpreted in a different way. However, we limited the interaction terms to type I which was aimed for parsimonious modeling and computation. For the type I interaction, the two unstructured effects $v_{i}$ and were interacting. Then the random effect $\delta_{it}$has its structure matrix as since both $v_{i}$ and don’t have a spatial or temporal structure. Thus, we assumed no strong spatial or temporal structures on the interaction term either and consequently $\delta_{it}$ had a zero-mean Gaussian prior. It was perhaps more sensible to assume the temporal parametric term to have a different coefficient for each province (i.e., random slope). So models 7-12 were specified as random slope modeling with a zero-mean Gaussian exchangeable prior on the temporal coefficient as $\beta_{1i}.$However we also would like to examine the spatial structure on the temporal coefficient and hence models 13-18 were assumed to have a BYM prior for each provincial slope as $\beta s_{1i}.$ Note that the prior distribution for non-spatial coefficient of fixed time effect was assumed as a zero-mean Gaussian with precision of 10^-6^ while all precision parameters for random effects were assumed on the log scale to have a log-Gamma distribution(1, 10^-5^) as the suggested default for INLA.

**S2: Hotspot analysis**

A number of diagnostic tools are available to evaluate the local anomalies. However, it is a natural idea to consider a hotspot as any isolated locations or geographically-bounded regions that display an excess of disease risk or incidence in a particular time. The excess of malaria risk can be examined by comparison with the expected rate. There are a number of ways to adjust for the baseline (see examples [3-5]), however a common practice for disease mapping [6] is to calculate the expected rate as $E_{it}=\frac{\sum_{i} \sum_{t} y_{it}}{\sum_{i} \sum_{t} pop_{it}}pop_{it}$, where$y_{it}$and$pop_{it}$are the number of malaria incidence and population at risk for each location and time. We would like to perform the analysis to find persistent hotspot over the study period, Then the expected rate used in the analysis was computed as $E_{i}=\frac{\sum_{i} \sum_{t} y_{it}}{\sum_{i} \sum_{t} pop_{it}}\overline{pop}_{i}$where $\overline{pop}$is the population at risk in each province averaged over the study period. So, an approach for space-time anomaly detection is to calculate $P(\mu_{i}/E_{i}>a)$, exceedance probability, from the number of estimates in the posterior sample which exceed a threshold [7, 8]. Usually the limit is assumed to be $a$=1 which means we apply the level of the expected rate as the baseline. The exceedance probability was calculated using the cumulative malaria cases over the study period in the likelihood with the BYM model for random effects and $E_{i}$as the offset.

**S3: Model computation**

To compute the malaria incidence in each province and time period, from a Bayesian perspective, the estimates can be viewed as missing data and an approach to impute the missing is to utilize the posterior predictive distribution which can produce both point estimates and uncertainty bands. The posterior predictive distribution of malaria cases is in the form:

$p(y_{it}|\tilde{y})=\int_{\Theta} p(y_{it}|\Theta,\tilde{y})p(\Theta|\tilde{y})d\Theta$ (1)

where$\tilde{y}$ and $\Theta$denotes the full data and parameter space used to fit the model.

Estimation from the predictive distribution can be generally obtained from converged posterior samplers using sampling-based algorithms, e.g., Markov chain Monte Carlo (McMC). However, there are a number of linear predictor specifications we would like to compare. With multi-dimensional model set up, the parameter space can expand quickly and demand computational resources exponentially. A more efficient approach to infer parameters in this context is the integrated nested Laplace approximation (INLA) [9]. With relative fast numerical routines for performing the above model specification and compatibility of our proposed model with INLA format, the model selection process was implemented in the framework of the Laplace approximation using the R-INLA package available from www.r-inla.org.

**S4: Plots of observed and predicted surveillance malaria cases of both *Plasmodium* species of hotspot provinces.**


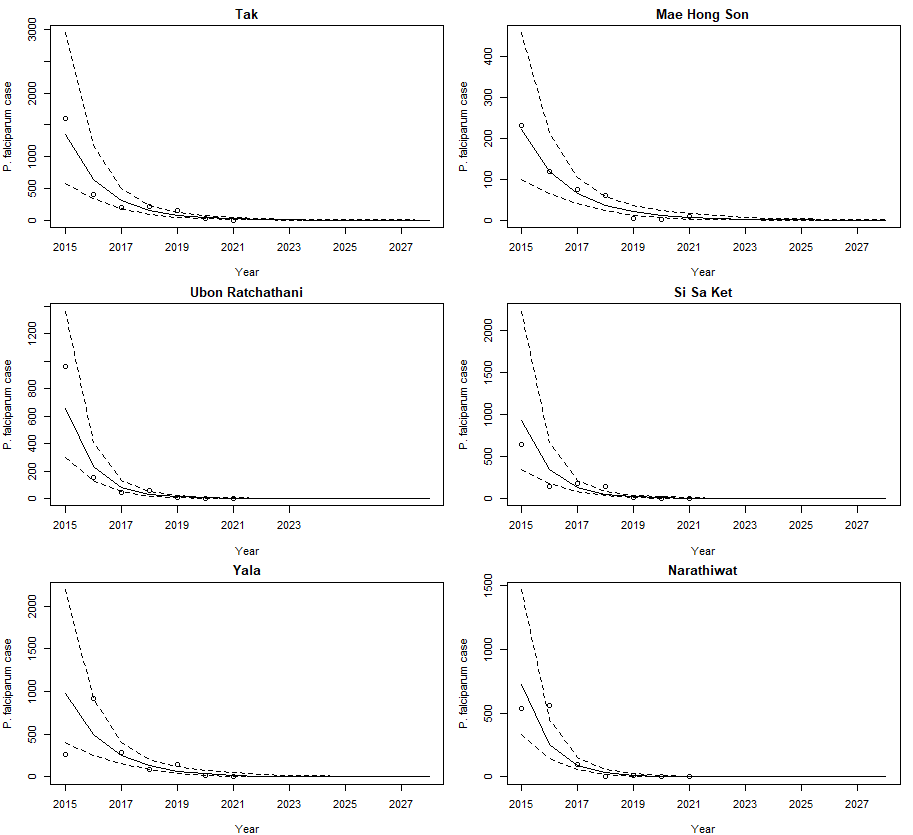


**Figure S1** Plots of *P. Falciparum* surveillance case estimates (solid line) with 95% credible band (dash line) of hotspot provinces. The dots are observed cases during the study period.


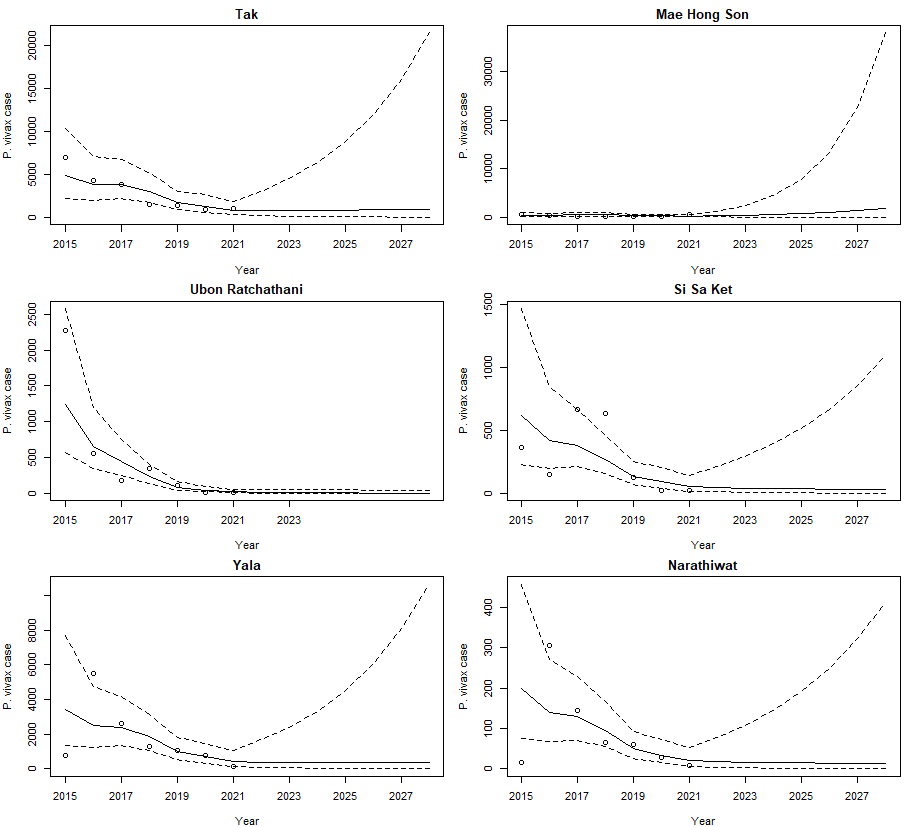


**Figure S2** Plots of *P. Vivax* surveillance case estimates (solid line) with 95% credible band (dash line) of hotspot provinces. The dots are observed cases during the study period.

Please be noted that variation in the predictive interval can be resulted in from a number of factors. One is from the variation within the data used to fit the model and then the variation is also reflected in the prediction. For example, Si Sa Ket had jumps in 2017-2018, which in turn yielded the estimate with a wider predictive interval than Ubon Ratchathani although they had an overall declining trend. However, for Mae Hong Son, the P. vivax trend was increased in later years and thus the model also produced a wide range in prediction.

**S5: Maps of upper and lower bound of predicted Thai malaria cases at provincial level**


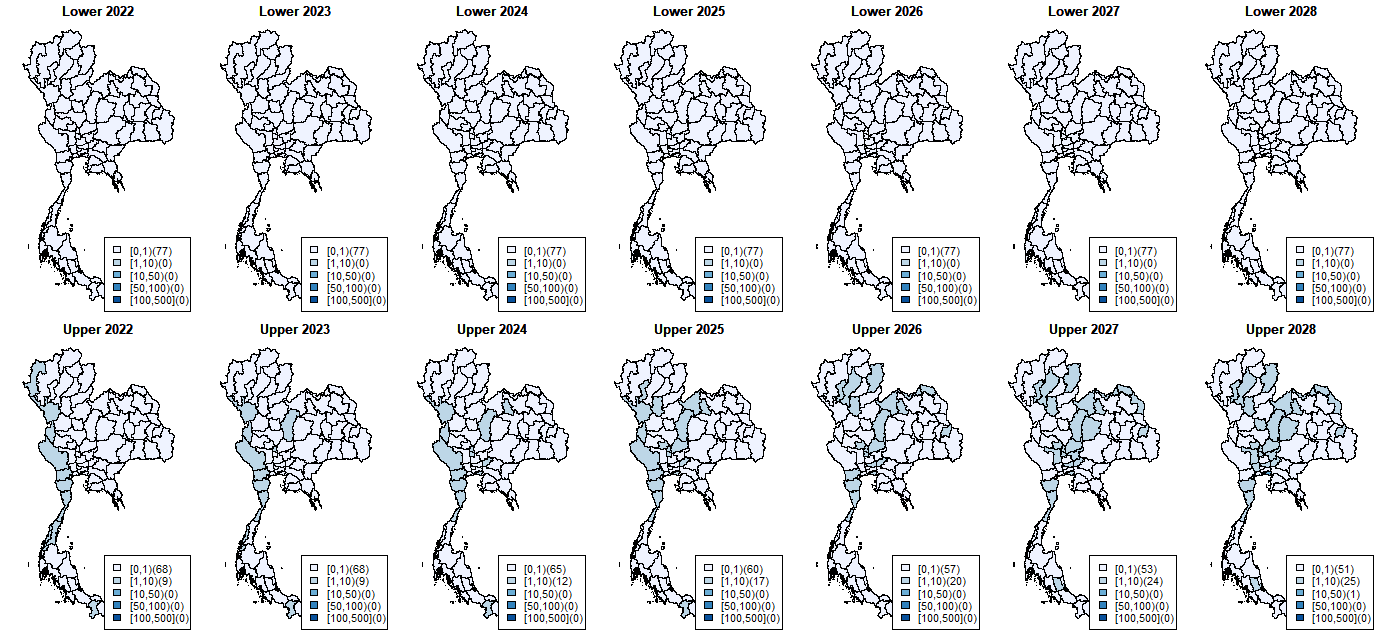


**Figure S3** Maps of predicted upper and lower bound of total *P. Falciparum* cases per 100,000 population at provincial level.


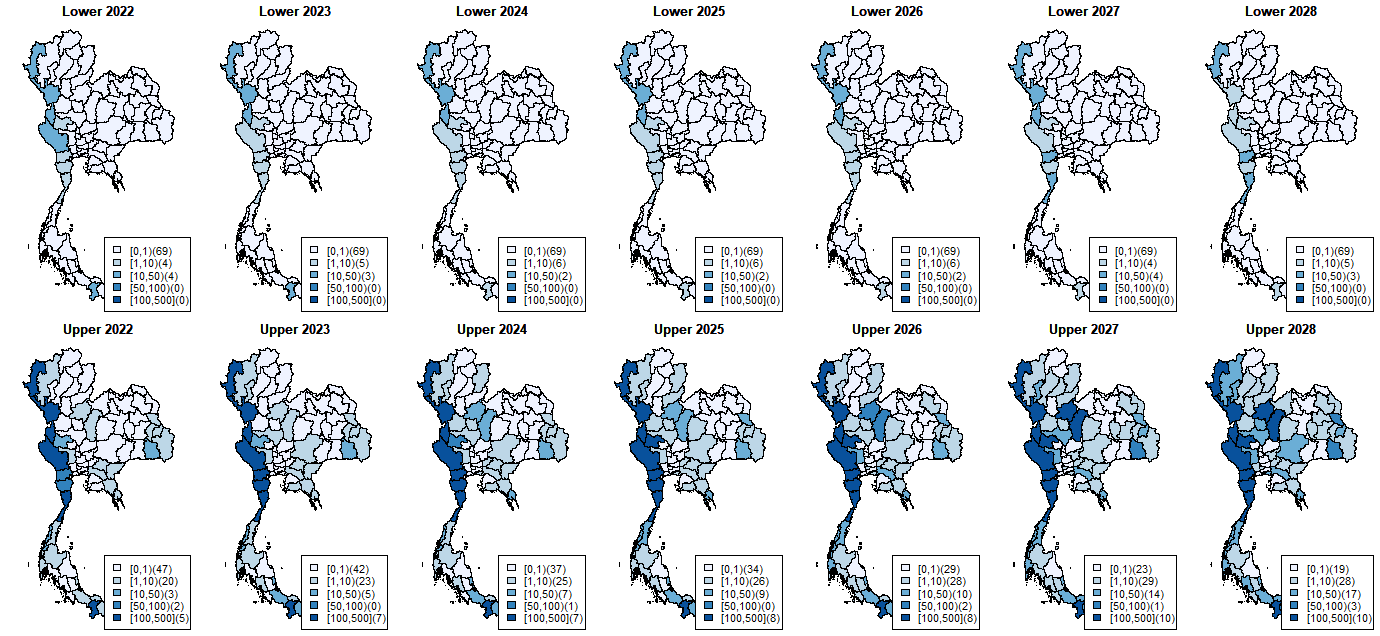


**Figure S4** Maps of predicted upper and lower bound of total *P. Vivax* cases per 100,000 population at provincial level.


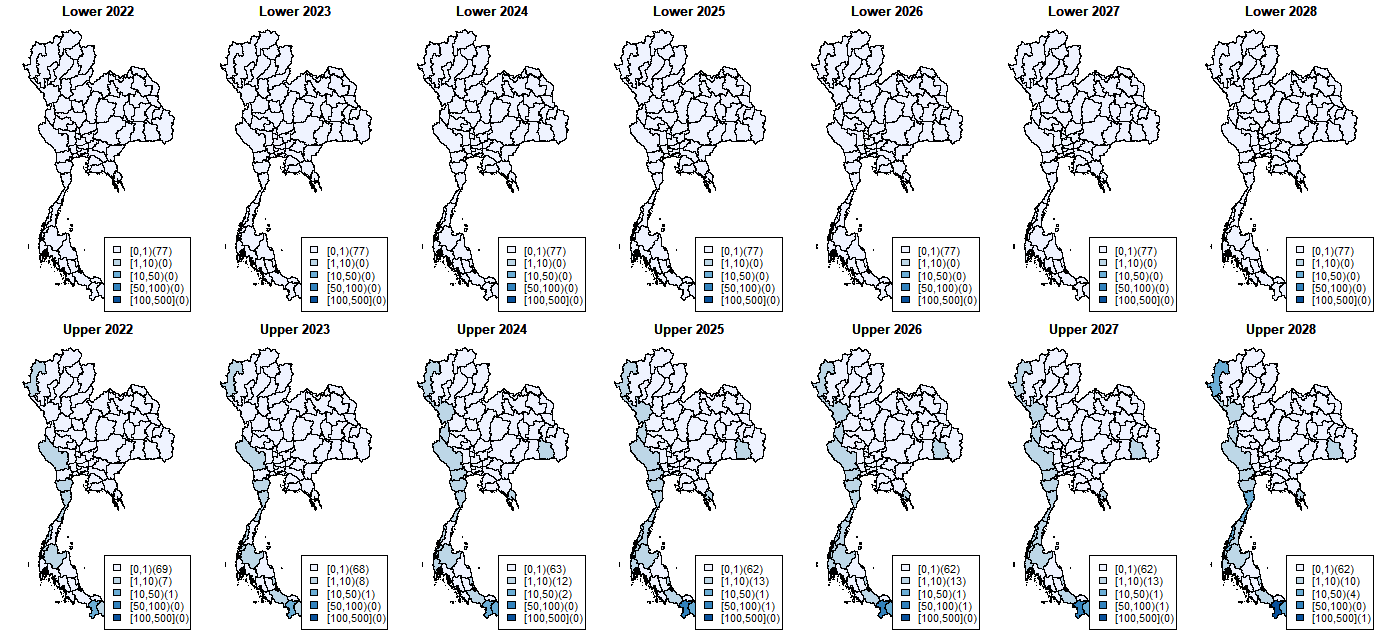


**Figure S5** Maps of predicted upper and lower of indigenous *P. Falciparum* cases per 100,000 population at provincial level.

**
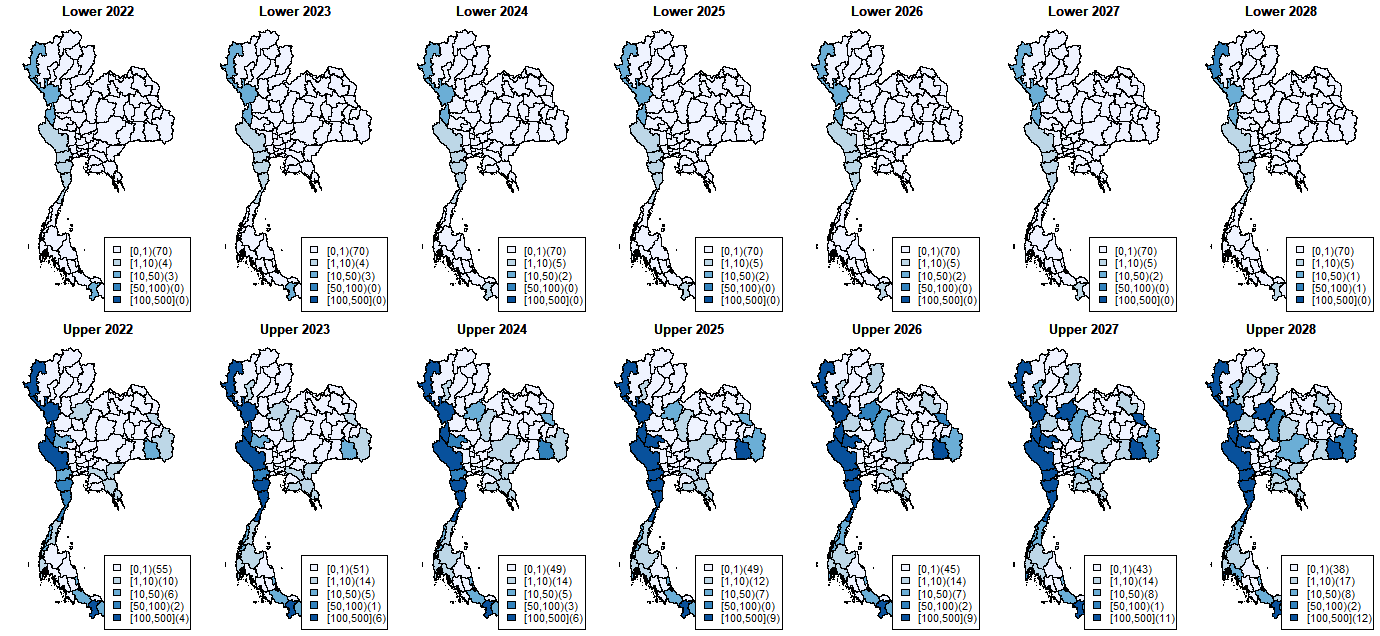
**

**Figure S6** Maps of predicted upper and lower bound of indigenous *P. Vivax* cases per 100,000 population at provincial level.

**S6: Estimates of dispersion parameter under model specifications and malaria case types**

| Model |  | Local Pf |  |  | Local Pf |  |  | Total Pf |  |  | Total Pv |  |
| --- | --- | --- | --- | --- | --- | --- | --- | --- | --- | --- | --- | --- |
|  | mean | lower | upper | mean | lower | upper | mean | lower | upper | mean | lower | upper |
| 1 | 1.094 | 0.793 | 1.557 | 1.287 | 1.019 | 1.652 | 0.533 | 0.413 | 0.700 | 0.768 | 0.640 | 0.929 |
| 2 | 1.108 | 0.831 | 1.524 | 1.190 | 0.994 | 1.326 | 0.532 | 0.412 | 0.696 | 0.770 | 0.634 | 0.928 |
| 3 | 1.108 | 0.833 | 1.503 | 1.285 | 1.018 | 1.656 | 0.534 | 0.414 | 0.700 | 0.766 | 0.641 | 0.931 |
| 4 | 1.096 | 0.820 | 1.507 | 1.283 | 1.012 | 1.644 | 0.531 | 0.412 | 0.696 | 0.767 | 0.639 | 0.927 |
| 5 | 1.107 | 0.833 | 1.511 | 1.003 | 0.730 | 1.497 | 0.533 | 0.413 | 0.698 | 0.765 | 0.641 | 0.937 |
| 6 | 1.092 | 0.824 | 1.519 | 0.641 | 0.335 | 1.346 | 0.532 | 0.412 | 0.695 | 0.772 | 0.653 | 0.931 |
| 7 | 0.824 | 0.586 | 1.201 | 0.924 | 0.704 | 1.246 | 0.313 | 0.231 | 0.432 | 0.509 | 0.409 | 0.643 |
| 8 | 0.816 | 0.570 | 1.208 | 0.901 | 0.688 | 1.210 | 0.314 | 0.231 | 0.439 | 0.511 | 0.409 | 0.647 |
| 9 | 1.102 | 0.819 | 1.522 | 0.897 | 0.680 | 1.200 | 0.330 | 0.241 | 0.466 | 0.482 | 0.385 | 0.608 |
| 10 | 0.715 | 0.504 | 1.033 | 0.917 | 0.665 | 1.294 | 0.331 | 0.241 | 0.466 | 0.482 | 0.386 | 0.610 |
| 11 | 0.814 | 0.571 | 1.196 | 1.318 | 1.027 | 1.737 | 0.028 | 0.011 | 0.083 | 0.133 | 0.059 | 0.301 |
| 12 | 0.799 | 0.570 | 1.133 | 0.918 | 0.701 | 1.237 | 0.006 | 0.001 | 0.061 | 0.515 | 0.410 | 0.647 |
| 13 | 8.532 | 6.993 | 10.537 | 0.953 | 0.669 | 1.443 | 0.314 | 0.232 | 0.437 | 0.509 | 0.409 | 0.645 |
| 14 | 0.953 | 0.673 | 1.387 | 0.906 | 0.679 | 1.237 | 0.315 | 0.232 | 0.440 | 0.513 | 0.414 | 0.645 |
| 15 | 2.151 | 2.021 | 2.177 | 2.565 | 2.157 | 2.987 | 0.344 | 0.258 | 0.483 | 0.486 | 0.390 | 0.616 |
| 16 | 0.729 | 0.503 | 1.091 | 0.926 | 0.748 | 1.166 | 0.332 | 0.244 | 0.467 | 0.482 | 0.389 | 0.609 |
| 17 | 8.547 | 7.008 | 10.537 | 0.010 | 0.003 | 0.061 | 0.010 | 0.008 | 0.010 | 2.186 | 1.909 | 2.526 |
| 18 | 0.953 | 0.685 | 1.361 | 0.907 | 0.689 | 1.225 | 0.316 | 0.232 | 0.442 | 0.510 | 0.408 | 0.644 |

**References**

1. Besag, J., J. York, and A. Mollié, *Bayesian image restoration, with two applications in spatial statistics.* Annals of the institute of statistical mathematics, 1991. **43**(1): p. 1-20.

2. Knorr‐Held, L., *Bayesian modelling of inseparable space‐time variation in disease risk.* Statistics in medicine, 2000. **19**(17‐18): p. 2555-2567.

3. Rotejanaprasert, C. and A. Lawson, *Bayesian prospective detection of small area health anomalies using Kullback–Leibler divergence.* Statistical methods in medical research, 2018. **27**(4): p. 1076-1087.

4. Rotejanaprasert, C. and A.B. Lawson, *A bayesian quantile modeling for spatiotemporal relative risk: an application to adverse risk detection of respiratory diseases in South Carolina, USA.* International journal of environmental research and public health, 2018. **15**(9): p. 2042.

5. Lawson, A.B., et al., *Handbook of spatial epidemiology*. 2016: CRC Press.

6. Aswi, A., et al., *Bayesian spatial and spatio-temporal approaches to modelling dengue fever: a systematic review.* Epidemiology & Infection, 2019. **147**.

7. Lawson, A.B., *Disease cluster detection: a critique and a Bayesian proposal.* Statistics in medicine, 2006. **25**(5): p. 897-916.

8. Lawson, A.B. and C. Rotejanaprasert, *Childhood brain cancer in Florida: a Bayesian clustering approach.* Statistics and Public Policy, 2014. **1**(1): p. 99-107.

9. Rue, H., S. Martino, and N. Chopin, *Approximate Bayesian inference for latent Gaussian models by using integrated nested Laplace approximations.* Journal of the royal statistical society: Series b (statistical methodology), 2009. **71**(2): p. 319-392.
